# Improving sustainable hydrogen production from green waste: [FeFe]-hydrogenases quantitative gene expression RT-qPCR analysis in presence of autochthonous consortia

**DOI:** 10.1186/s13068-021-02028-3

**Published:** 2021-09-16

**Authors:** M. Arizzi, S. Morra, G. Gilardi, M. Pugliese, M. L. Gullino, F. Valetti

**Affiliations:** 1 Department of Life Sciences and Systems Biology, University of Torino, Via Accademia Albertina 13, 10123 Torino, Italy; 2 Centre of Competence for Innovation in Agro-Environmental Field (Agroinnova) and DiSAFA, University of Torino, Largo Paolo Braccini 2, 10095 Grugliasco, TO Italy; 3 AgriNewTech Srl, Via Livorno 60, 10140 Torino, Italy; 4 Present Address: Faculty of Engineering, University of Nottingham, Nottingham, UK; 5 Present Address: Acea Engineering Laboratories Research Innovation SpA, Roma, Italy

**Keywords:** [FeFe]-hydrogenase, Reverse transcriptase quantitative PCR (RT-qPCR), Dark fermentative hydrogen-producing bacteria (dHPB), Bio-hydrogen, *Clostridium*

## Abstract

**Background:**

Bio-hydrogen production via dark fermentation of low-value waste is a potent and simple mean of recovering energy, maximising the harvesting of reducing equivalents to produce the cleanest fuel amongst renewables. Following several position papers from companies and public bodies, the hydrogen economy is regaining interest, especially in combination with circular economy and the environmental benefits of short local supply chains, aiming at zero net emission of greenhouse gases (GHG). The biomasses attracting the largest interest are agricultural and urban green wastes (pruning of trees, collected leaves, grass clippings from public parks and boulevards), which are usually employed in compost production, with some concerns over the GHG emission during the process. Here, an alternative application of green wastes, low-value compost and intermediate products (partially composted but unsuitable for completing the process) is studied, pointing at the autochthonous microbial consortium as an already selected source of implementation for biomass degradation and hydrogen production. The biocatalysts investigated as mainly relevant for hydrogen production were the [FeFe]-hydrogenases expressed in Clostridia, given their very high turnover rates.

**Results:**

Bio-hydrogen accumulation was related to the modulation of gene expression of multiple [FeFe]-hydrogenases from two strains (*Clostridium beijerinckii* AM2 and *Clostridium tyrobutyricum* AM6) isolated from the same waste. Reverse Transcriptase quantitative PCR (RT-qPCR) was applied over a period of 288 h and the RT-qPCR results showed that *C. beijerinckii* AM2 prevailed over *C. tyrobutyricum* AM6 and a high expression modulation of the 6 different [FeFe]-hydrogenase genes of *C. beijerinckii* in the first 23 h was observed, sustaining cumulative hydrogen production of 0.6 to 1.2 ml H_2_/g VS (volatile solids). These results are promising in terms of hydrogen yields, given that no pre-treatment was applied, and suggested a complex cellular regulation, linking the performance of dark fermentation with key functional genes involved in bio-H_2_ production in presence of the autochthonous consortium, with different roles, time, and mode of expression of the involved hydrogenases.

**Conclusions:**

An applicative outcome of the hydrogenases genes quantitative expression analysis can be foreseen in optimising (on the basis of the acquired functional data) hydrogen production from a nutrient-poor green waste and/or low added value compost, in a perspective of circular bioeconomy.

## Background

The exploitation of wastes to recover new materials, energy and fuels is a key point of circular economy. The biochemical processes are among the most effective strategies to efficiently maximise this approach, and thus the strong and intertwined link between circular economy and bioeconomy. The greenhouse gases (GHG) neutrality is a natural consequence of the equilibrium among the biochemical processes involved in bioeconomy. In the perspective of a constantly growing energy demand, maximising the efficiency of energy recovery from waste and limiting GHG at the same time is an obligate pathway to make the process sustainable and economically feasible. In a biological approach the focus is on recovering the reducing equivalents from organic compounds, avoiding C and N loss under the form of GHG. Also, the efforts should focus to the production of bio-fuels which does not produce GHG (or are GHG neutral). In this perspective, bio-hydrogen is a very promising bio-fuel and a detailed research in biochemistry and biotechnology is the tool to reach the envisaged optimisation of the recovering processes.

Hydrogen can be produced by either biological, electrochemical or thermochemical processes. Compared with other biological hydrogen production processes, dark fermentation appears to be the most appealing method for the following reasons: (i) it can continually produce H_2_ even in the absence of light; (ii) it can use a variety of low-value waste as raw materials such as organic wastewater and waste biomass [1, 2]; (iii) fermentation by-products with alternative value include butyric, lactic and acetic acid; and (iv) the bacteria used are anaerobic, thus costly sparging with O_2_ would be spared.

To date, research has been focused on reactor optimisation and fermentation conditions [3–7] while the microbial community structure and how this impacts on H_2_ production are still topics to be elucidated in more detail, although interest on these aspects is recently growing [7–10].

Dark fermentative hydrogen-producing bacteria (dHPB) such as *Clostridium*, *Ethanoligenens*, *Enterobacter* and *Bacillus* have been isolated from bioreactors and natural environments and previous studies have shown that culture conditions or operating parameters can significantly affect cell growth and hydrogen production [11]. The identification and characterisation of highly efficient hydrogen-producing bacteria is a very crucial point in applicative term. In order to efficiently operate biohydrogen-producing dark fermentation processes, or adjust parameters upon malfunctions, it is important to understand how the system works in real mixed consortia and in applicative condition. In a hydrogen-fermenting bioreactor, where the community structure, including microorganisms other than hydrogen producers, can change substantially over time [12] functional detection would provide a tool for bioprocess monitoring. Quantification of the main producers, monitoring the expression of their key genes for hydrogen production and monitoring the community structure during operation is essential in order to understand what kind of community changes are linked to changes in the bioreactor operation [13].

Hydrogenases are enzymes known to be responsible for H_2_ production. They can be divided into three groups based on their metal content in the H_2_-activating sites: [Fe]-hydrogenases, [FeFe]-hydrogenases, and [NiFe]-hydrogenases.

The [FeFe]-hydrogenases are very efficient hydrogen-producing biocatalysts with a turnover frequency of up to 10^4^ s^−1^ [14]. They are widespread among bacteria and highly represented in Gram-positive *Clostridium spp.* [13]. These bacteria are often considered to be the main group of H_2_ producers in mesophilic, hydrogen-fermenting bioreactors, which makes the [FeFe]-hydrogenase a valuable target for analysis.

[FeFe]-hydrogenases are monomeric, dimeric, trimeric or tetrameric enzymes with a highly modular structure; they have at least a catalytic active H-domain and may present other accessory domains, which are responsible for the large biodiversity in this class of enzymes [14, 15].

In addition, several microorganisms possess more than one [FeFe]-hydrogenase annotated gene-encoding enzymes belonging to different subgroups. This redundancy has only partially been explored [16] and, while it is expected to imply various roles in the cell growth and energetics for the different genes, depending on the time of expression and modulation, the correlation is still elusive.

So far, only few members of the [FeFe]-hydrogenase class have been studied and characterised in details: these enzymes belong to species of the genus *Desulfovibrio*, especially *D. desulfuricans* (DdH) [17]; species of the genus *Clostridium* especially *C. pasteurianum,* with the two variants CpI [18] and CpII [16] and *C. acetobutylicum* (CaHydA) [19] as well as some green algae like *Chlamydomonas reinhardtii* (CrHydA1) [20].

It is indeed becoming clearer that the study of new uncharacterised [FeFe]-hydrogenases might contribute to a broader knowledge of molecular mechanism of H_2_ production and to the isolation of novel and improved biocatalysts with unexpected features, such as resistance to oxygen damage and quick recovery of full activity upon changes of redox state, gas-sensing-role, high electron-exchange efficiency for biotechnological exploitation in bio-hybrid systems (i.e. microbial fuel cells and enzyme-modified electrodes) [21–32].

This research had multiple aims: firstly, it aimed at the isolation, identification and characterisation of efficient hydrogen-producing bacteria (with their [FeFe]-hydrogenases as enzyme catalysts) that may be suitable candidates as inoculum on waste biomasses for hydrogen production. In this perspective, the waste matrix and autochthonous consortium used has already demonstrated to be able to sustain hydrogen and methane production [33], but here a detailed description is provided. Secondly, by performing the extensive investigation of the time and mode of genes expression of the newly identified [FeFe]-hydrogenases in a mixed culture naturally present in waste biomass, the work presented here grants data for a better knowledge of the mechanisms of bio-hydrogen production in real dark fermentation systems and an additional information on the fundamental biochemical keys to tune and improve the performances. As a third and more applicative aspect, this work can also contribute to consolidate the use of green waste as a substrate for bio-hydrogen and bio-methane production (and only the resulting digestate for compost production), rather than address the largely available green waste to direct composting, which might result in net loss of recoverable energy and in the typical emission, during composting, of GHG such as methane and N_2_O [34].

## Results and discussion

### Isolation and microbial community analysis of hydrogen-producing bacteria from green waste biomass

The cultivable microbiota was analysed in an autumnal green waste biomass (named *Mix*) that is usually sent to standard composting processes. Given the previous evidence that this kind of biomass alone can sustain dark fermentation, producing hydrogen at appreciable levels [33], the interest was to grant a detailed characterisation of the microbiological and biochemical aspects in order to complete the analysis.

All the isolated microorganisms were able to grow in anaerobic conditions and among these 36% were oxygen-tolerant. The Gram staining showed that all the isolated were Gram + ; 26% of the isolated microorganisms were cocci (all of these were oxygen tolerant) and 74% bacilli (among these 56% oxygen-tolerant and 46% oxygen-sensitive).

Subsequently, the isolates were classified in further detail on a molecular basis, by means of 16S rRNA-encoding genes restriction fragment length polymorphism (RFLP) analysis. The 16S rRNA-encoding gene was amplified by PCR from each isolated strain and digested with four restriction endonucleases (*Hae*III, *Alu*I, *Hha*I, *Taq*I).

**Fig. 1:**
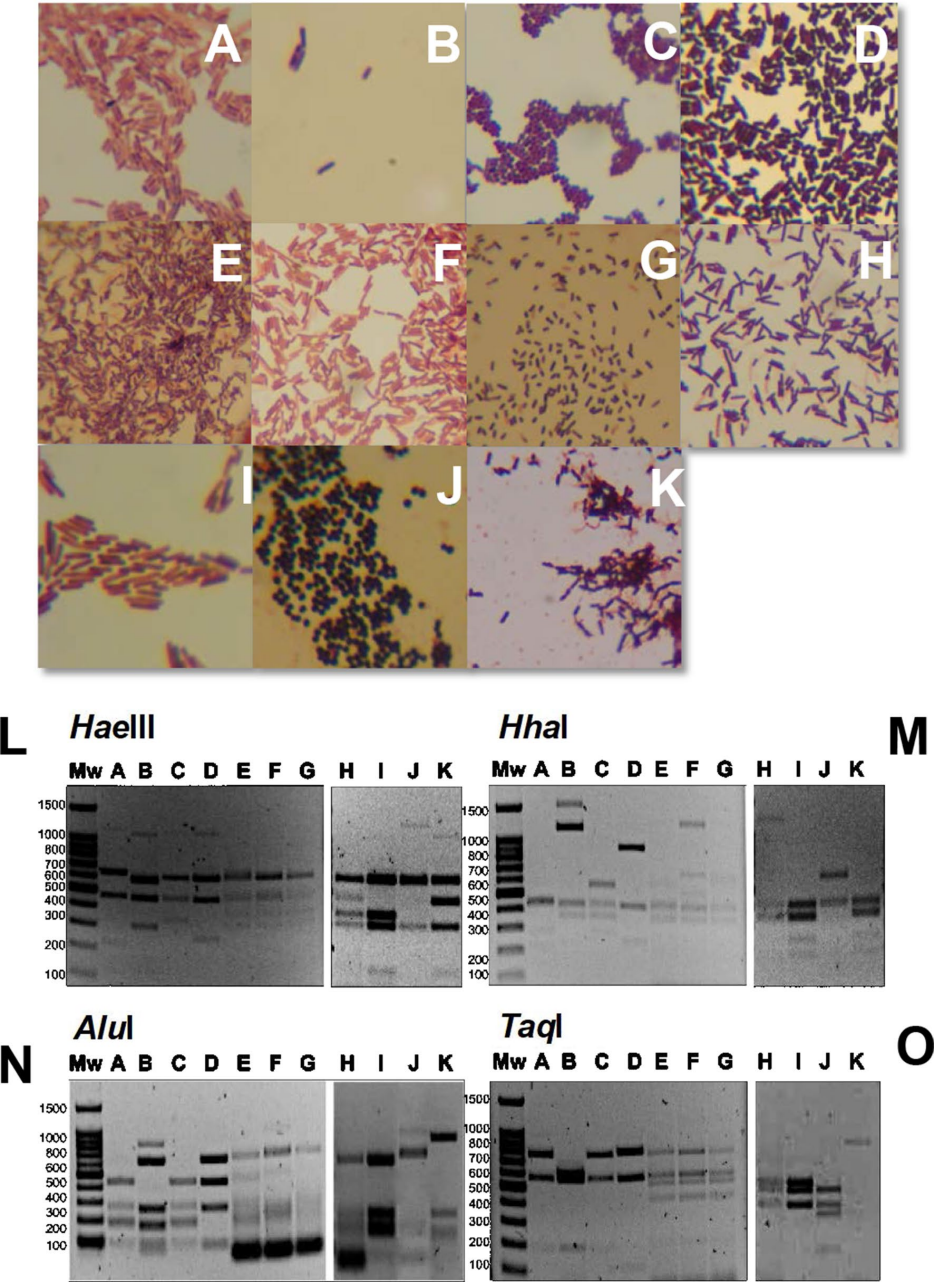
Morphologic and molecular characterisation of 11 different group type of bacteria isolated from green waste biomass. **A**–**K** Gram staining of the species. The bacteria shown are: *Lactobacillus mucosae* (**A**), *Clostridium beijerinckii* (**B**), *Pediococcus acidilactici* (**C**), *Clostridium tyrobutyricum* (**D**), *Lactobacillus fermentum* (**E**), *Lactobacillus acidipiscis* (**F**), *Lactobacillus collonoides* (**G**), *Lactobacillus sp.* (**H**), *Bacillus ginsengihumi* (**I**), *Staphylococcus simulans* (**J**), *Bacillus licheniformis* (**K**). **L**–**O** 16S rRNA-encoding genes RFLP analysis of the **A** to **K** isolated bacteria, using different restriction enzymes: *Hae*III (**L**), *Hha*I (**M**), *Alu*I (**N**), *Taq*I (**O**)

The morphological features, growth ability and RFLP analysis allowed the subdivision of all the isolated strains into 11 different group types from A to K (Fig. 1). The identified species (on the basis of the sequencing of 16S rRNA-encoding genes with the related uncertainties) are *Clostridium beijerinckii*, *Clostridium tyrobutyricum*, *Pediococcus acidilactici*, *Bacillus ginsengihumi*, *Bacillus licheniformis*, *Staphylococcus simulans, Lactobacillus mucosae, Lactobacillus fermentum, Lactobacillus acidipiscis, Lactobacillus collonoides and Lactobacillus sp*.

The identified bacteria, *Clostridium sp.*, *Lactobacillus sp*., *Pediococcus sp*., *Staphylococcus sp.* and *Bacillus sp.* are commonly found in soil or fermentable materials [35–37].

**Fig. 2:**
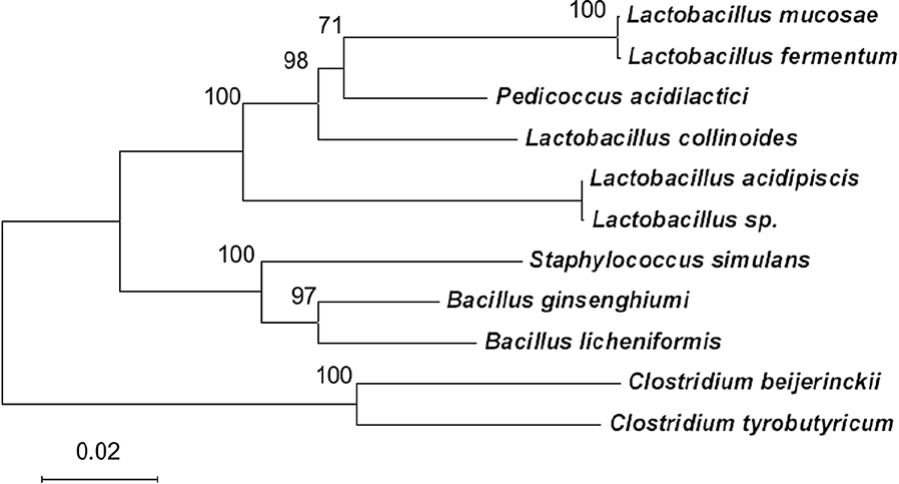
Phylogenetic tree based on 16S rRNA-encoding gene sequences of the isolate bacteria from green waste. The tree was constructed using the neighbour-joining algorithm. Numbers on the tree refer to bootstrap values on 1000 replicates. The bar indicates a 2% estimated difference in nucleotide sequences. Analogous phylogenetic analysis of *Clostridium beijerinckii* compared to other standard Clostridia and of *Bacillus ginsengihumi* related to reference strains can be found in [37] for Bacilli and in [2] for Clostridia. The reference numbers for the 16S rRNA-encoding gene sequences are listed below (group A to K as specified in Fig. 1 caption): group A: MZ054377, group B: MZ054378, group C: MZ054379, group D: MZ054380, group E: MZ054381, group F: MZ054382, group G: MZ054383, group H: MZ054384, group I: MZ054385, group K: MZ054386

The microbial diversity found in green waste biomass is illustrated through a phylogenetic analysis of 16S rRNA-encoding genes sequences (Fig. 2).

The *consortium* was composed of two macro-groups: pro-biotic non-hydrogen-producing bacteria (belonging to the genera *Lactobacillus* and *Bacillus*) and hydrogen-producing bacteria (belonging to the genus *Clostridium*). Recent interest has been given to the interplay of hydrogen producers and non-hydrogen producers in mixed consortia degrading agricultural wastes such as wheat and reed straw [38, 39], and other available wastes such as sugar bagasse [9], highlighting the importance of native waste-associated consortia in regulating the hydrogen production, even outperforming selected inoculum taken from anaerobic digestion plants. The presence of autochthonous *Lactobacillus* and *Bacillus* strains in the *Mix* sample is in line with previously reported analysis of mixed consortia [38–40] and their effect in hydrogen-producing digester. The autochthonous consortium was challenged here with a bio-augmentation strategy, exploiting the good performances already proved by the autochthonous microbiota on similar and very poor substrates in previous analysis [33] and testing a possible positive synergy between the consortium and the added inoculum.

The ability to produce H_2_ was assayed to investigate the direct involvement in H_2_ production of each species. As expected, only two species of the genus *Clostridium* were able to produce H_2_: *Clostridium beijerinckii* and *Clostridium tyrobutyricum.* Various strains of *C. beijerinckii* were isolated from several sources [2, 29, 35, 41] and *C. tyrobutyricum* has mainly been used in fermentation to produce butyric acid and only limitedly in hydrogen production [42, 43].

However, the hydrogen-producing bacteria isolated here are different strains from those annotated in databases. The 16S rRNA-encoding gene sequence of *C. beijerinckii* displayed a 100% of identity with the 16S rRNA-encoding gene of the *C. beijerinckii* stains annotated in databases, but a further analysis of the sequences of the six annotated hydrogenases for this species showed few differences, hence the name *C. beijerinckii* AM2*.* The 16S rRNA-encoding gene of *C. tyrobutyricum* showed a 99% of identity with two of the *C. tyrobutyricum* stains annotated in databases, therefore named *C. tyrobutyricum* AM6*.* The two novel strains isolated were found to be two efficient hydrogen producers: *C. beijerinckii* AM2 produced 299.4 ± 2 mL of H_2_ per g of glucose and *C. tyrobutyricum* AM6 released 246 ± 7 mL of H_2_ per g of glucose when tested as individual isolates.

The maximum hydrogen yields of the strain isolated from green waste were higher than those previously reported in the literature for other hydrogen-producing bacteria like *C. perfringens* 130 ± 3 mL of H_2_ per g of glucose [35]; *C. butyricum* 136 ± 5 mL of H_2_ per g of glucose [35]; *C. diolis* 150 mL of H_2_ per g of glucose [44]; *C. beijerinckii Fanp3* 231 mL of H_2_ per g of glucose [29] and *C. tyrobutyricum* JM1 223 ml of H_2_ per g of hexose [42].

Therefore, these novel strains (*C. beijerinckii* AM2 and *C. tyrobutyricum* AM6) were selected for the identification of their [FeFe]-hydrogenases and quantification of expression levels.

### [FeFe]-hydrogenase genes of *C. beijerinckii* AM2 and *C. tyrobutyricum* AM6

Fermentative hydrogen production in the genus *Clostridium* is related with the activity of the [FeFe]-hydrogenases, these enzymes use protons as the final electron acceptors in the cellular energy metabolism. In general the transcription of [FeFe] hydrogenases encoding genes has been previously applied also in meta-trascriptomic approaches to estimate the hydrogen-producing bacterial consortium in selected soils [45].

*C. beijerinckii* has six genes encoding for different [FeFe]-hydrogenases, 4 monomeric and 2 heterotrimeric and a gene for a [NiFe]-hydrogenase [15]. *C. tyrobutyricum* has one gene encoding for a monomeric [FeFe]-hydrogenase that has been linked to hydrogen production [42] and that until 2016 has been considered as the only [FeFe]-hydrogenase-encoding gene in this strain. Only recently [43] new hypothetical sequences assigned to hydrogenase-encoding genes have been proposed for some specific *C. tyrobutyricum* strains, but these latter sequences were not considered in this work.

Each selected gene encodes a protein belonging to a specific and diverse modular structure type and phylogenetic cluster according to a previously reported classification [15].

The 6 *hyd* genes of *C. beijerinckii* are: *Cbei_1773* (encoding for a protein with structure type M2c and cluster A5), *Cbei_1901* (encoding for a protein with structure type M2a and cluster B2), *Cbei_0327* (encoding for a protein with structure type M3a and cluster B3), *Cbei_4000* (encoding for a protein with structure type M2b and cluster A2), *Cbei_3796* (encoding for a protein subunit with structure type TR (M2) and cluster A3) and *Cbei_4110* (encoding for a protein and catalytic subunit with structure type structure type TR (M3) and cluster A8). The gene *hyd* of *C. tyrobutyricum* (*Ctyr_hydA*) encodes for a protein with structure type M3 and cluster A2. All the gene products are likely to be cytoplasmic, with the exception of those belonging to cluster A3 (structure type TR (M2)). Interestingly enough, the *C. beijerinckii* [FeFe]-hydrogenase enzymes have received little attention in the past and even recently the trascriptomics analysis were more focused on other metabolic peculiarities of this strain [46–48], with some indication on genes involved in the hydrogen metabolism only reported in the paper of Patakova et al., 2019 [46].

Although recent works from our group highlighted the peculiarity of at least one of these enzymes [27] and demonstrated the relevance and complexity of their expression in pure cultures [35], the study of their genes expression modulation with quantitative methods has never been performed before. The *C. tyrobutyricum* [FeFe]-hydrogenase protein selected and targeted in this study is known to be involved in H_2_ production [42], but no studies are reported on the gene expression analysis of this enzyme.

The activity of [FeFe]-hydrogenases can be regulated at metabolic level through regulatory controls at transcriptional level [13].

It is already known that Clostridia present more than one gene encoding for [FeFe]-hydrogenases, which could be subjected to different expression modulation [49]; one aim of this research was the identification of the relationship between the performance of dark fermentation on a real and nutrient-poor green waste, already containing a mixed microbial consortium, and the expression of [FeFe]-hydrogenase genes, by measuring on the same samples the transcription levels of the selected genes and the hydrogen produced.

A previous analysis on the relationship between hydrogen production and [FeFe]-hydrogenases gene expression in *C. beijerinckii* was performed only in pure cultures and based on semi-quantitative estimate of transcripts [35].

RT-qPCR is a powerful and widely used techniques for detecting and quantifying specific gene expression in vitro. This technique has already been used for quantification of [FeFe]-hydrogenases and has proven to be an accurate assay for quantification of hydrogenase expression levels either in single or mixed bacterial cultures. Nonetheless, the previous studies of hydrogenases genes expression developed in various microorganisms have focused on a single [FeFe]-hydrogenase gene only in bioreactors or multiple [FeFe]-hydrogenase genes expression analysis only in pure cultures [13, 35, 49–51] or, when in syntrophic co-cultures, only under very controlled and simplified conditions and in rich media [50, 52]. Other studies performed on real complex matrices only provided an estimate of the hydrogen-producing consortium composition by meta-trascriptomic analysis and were not quantitative [45].

Here, the gene expression modulation in time of the seven genes encoding for [FeFe]-hydrogenase enzymes with different structure types was investigated by RT-qPCR in the hydrogen-producing real biomasses waste, hence in presence of the autochthonous strains, both for the non-producers and the hydrogen-producing strains, either with applied bio-augmentation or not.

The *recA* single copy gene was used to monitor the total bacteria in the medium by quantitative PCR (qPCR). As a population indicator, this gene is a better indicator than the multiple copy 16S rRNA-encoding gene (up to 14 copies in *C. beijerinckii*) that might otherwise compromise the interpretation of the quantitative results, also when the whole genome sequence is unknown, such as in the case of *C. tyrobutyricum*, in which the estimation of the correct copy number can be problematic [49].

In addition, *recA* gene expression (cDNA) was used as the internal reference gene for RT-qPCR, according to the available literature on Clostridia [49, 53, 54], to monitor the active bacterial population, since the *recA* expression profile shows the microbial population that is viable and with transcriptional capacity [49, 53, 54]. Also in other bacterial species recA scored as the best in expression stability ranking of the candidate reference genes according to 4 software out of 5 (BestKeeper, NormFinder original softwares and Delta CT and RefFinder analysis) as reported in a recent paper exploring reference genes for gene expression studies using reverse transcription quantitative real-time PCR [55].

Strains specific primers were designed (see Materials and methods) and the specificity was tested experimentally. The maximal *recA* expression level (Fig. 4A) was detected at 23 h and then the amount of viable bacteria decreased at 37 h.

**Fig. 3:**
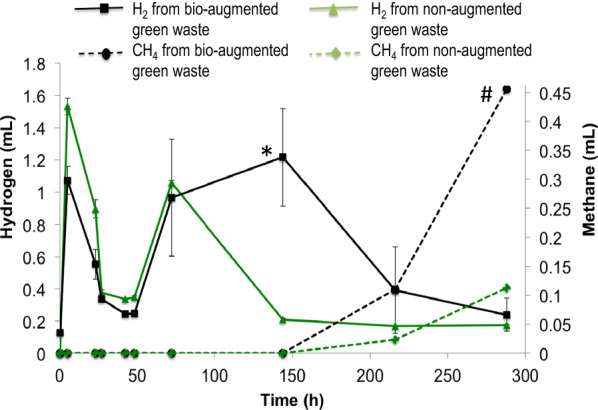
Cumulative hydrogen (full line, referred to left y-axis) and methane (dashed line; referred to right y-axis) production curves during dark fermentation of both hydrogen-producing *C. beijerinckii* AM2 and *C. tyrobutyricum* AM6 bio-augmented (black line) and non-augmented (green line) agriculture waste biomass. The values refer to 5 gr of green waste (as detailed in the Materials and Methods section). The VS for this quite heterogeneous material was between 250 and 500 gr VS/kg, therefore the cumulative hydrogen production is of 0.6–1.2 ml H_2_/gr VS, while for methane the cumulative value is of 0.18–0.36 ml CH_4_/gr VS. Significant differences were determined with a Student's t-test, *p < 0.05, #*p* < 0.01, ##*p* < 0.001 of bio-augmented *versus* non-augmented for each timepoint

**Fig. 4:**
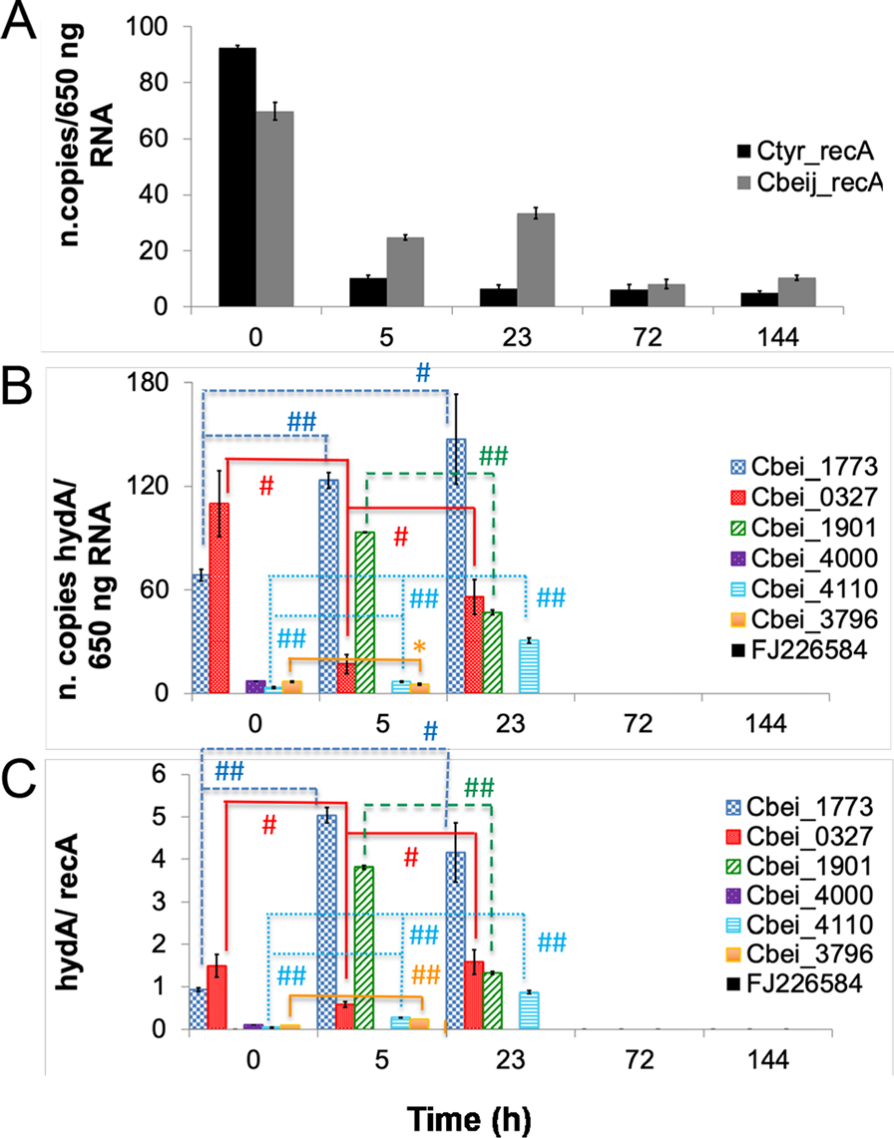
Relationship between *Clostridium beijerinckii* AM2 and *Clostridium tyrobutyricum* AM6 growth and [FeFe]-hydrogenase transcription levels in mixed culture present in agriculture waste biomass. **A** *recA* genes expression profiles from *Clostridium beijerinckii* AM2 and *Clostridium tyrobutyricum* AM6. **B** *Clostridium beijerinckii* AM2 and *Clostridium tyrobutyricum* AM6 [FeFe]-hydrogenase genes expression profiles during dark fermentation. **C** *Clostridium beijerinckii* AM2 and *Clostridium tyrobutyricum* AM6 [FeFe]-hydrogenase transcription levels normalised on *recA*. Significant differences in time were determined with a one-way ANOVA with a post hoc Student's t-test, *p < 0.05, #*p* < 0.01, ##*p* < 0.001 in the pairs indicated in figure

After checking the basal expression level of the reference gene *recA*, in order to ensure that hydrogen production and transcriptional levels of gene expression were high enough to be detected and properly quantified during the batch dark fermentation process, *C. beijerinckii* AM2 10^5^ CFU/mL and *C. tyrobutyricum* AM6 10^6^ CFU/mL were inoculated in the spring green green waste. Methane and hydrogen productions (Fig. 3), metabolically active bacteria *C. beijerinckii* AM2 and *C. tyrobutyricum* AM6 as well as 7 *hyd* genes transcript levels of *C. beijerinckii* AM2 and *C. tyrobutyricum* AM6 (Fig. 4) were analysed over a period of 288 h.

### Gas production in green waste during dark fermentation with and without bio-augmentation with *C. beijerinckii* AM2 and *C. tyrobutyricum *AM6

The cumulative curves of gas productions are summarised in Fig. 3, showing the comparison of green waste biomass with *C. beijerinckii* AM2 and *C. tyrobutyricum* AM6 bio-augmentation as well as green waste biomass alone (reported, respectively, in black and in green).

The hydrogen production curves from both the waste sample with and without bio-augmentation showed a similar trend with a production spike after 5 h, then hydrogen release decreased after 42 h to increase again after 72 h. A decrease in hydrogen production was observed in the non-bio-augmented sample after 144 h, whereas the waste which underwent bio-augmentation maintained a steady and sustained hydrogen production value, decreasing only after 216 h due to a rise of CH_4_ gas. The *C. beijerinckii* AM2 and *C. tyrobutyricum* AM6 enrichment did not cause an exponential increase in hydrogen production, but after 150 h of growth, the bio-augmented batch cultures produced a sixfold higher hydrogen amount, keeping it for a longer period than those from the non-augmented batch.

Methane productions from the bio-augmented sample and the non-augmented waste had a similar trend and methane production is revealed after 216 h; hydrogen and methane productions were mutually exclusive as expected [33]. The comparison of the trends in hydrogen and methane production in bio-augmented and non-augmented samples highlights that the bio-augmented sample is a good indicator of the spontaneous processes occurring in the waste biomass and it suggest that the autochthonous microbial consortium is not significantly inhibited upon addition of an inoculum, but a positive synergy can be implemented between the autochthonous consortium and the inoculum. The advantage of a higher hydrogen concentration, as the one observed in the bio-augmented sample between 72 and 216 h, resulting in a sixfold higher hydrogen amount, points to a possible further optimisation for applicative purposes. A further enhancement of hydrogen production could be achieved by pre-treating the waste in order to inhibit methanogenesis which is obviously here competing with hydrogen accumulation, as reported in Fig. 3. The project supporting the research (funded by EU to enhance bioeconomy and circular economy in the Piedmont region) aimed at exploring the potential used of untreated wastes, so no chemical or thermal treatment was applied to hinder methane production.

In the waste biomass bio-augmented with *C. beijerinckii* AM2 and *C. tyrobutyricum* AM6, the relation between hydrogen production and the modulation of the seven [FeFe]-hydrogenase genes expression was investigated during dark fermentation after set-up of the analysis method by RT-qPCR (Fig. 4).

*C. beijerinckii* and *C. tyrobutyricum recA* expressions are indicators of viable and metabolically active populations. Active cells contain more RNA than inactive cells. Predominance of metabolically active *C. beijerinckii* AM2 and *C. tyrobutyricum* AM6 species was estimated in waste biomass based on RT-qPCR monitoring of *recA* gene during dark fermentation, and *C. beijerinckii* AM2 prevails over *C. tyrobutyricum* AM6 although *C. tyrobutyricum* AM6 was added in larger quantities at the beginning of the process (Fig. 4A).

*C. tyrobutyricum* AM6 [FeFe]-hydrogenase gene expression was undetectable during the whole analysis, even when a second couple of primers were used to enhance amplification efficiency (Ctyr_hydA see Material and method section). As suggested by the *recA* expression profile (Fig. 4A), this bacterium, isolated from autumnal agriculture waste biomass, was likely to be in a quiescent spore form and not able to sustain an active grow on the green waste biomass under the tested conditions. Although the growth rate were similar in pure cultures for the two tested strains, the competition favoured the growth of *C. beijerinckii* AM2 on the green waste used.

All the *C. beijerinckii* AM2 [FeFe]-hydrogenase genes are expressed with a variable modulation in the first 23 h of the fermentative process, while after 72 h no [FeFe]-hydrogenase transcriptional activity was detected (Fig. 4B and C).

*C. beijerinckii* AM2 [FeFe]-hydrogenase transcription levels were detected from the beginning of the process. High levels of expression for [FeFe]-hydrogenase genes were observed before optimal hydrogen productions according to the literature [56].

At time 0 (inoculum addition) *Cbei_1773* and *Cbei_0327* genes were expressed, respectively, 1- and 1.5-fold more than the genes encoding for the monomeric *Cbei_4000*, the membrane-bound *Cbei_3796* and the catalytic subunit alpha *Cbei_4110* of the heterotrimeric [FeFe]-hydrogenases; *Cbei_1901* transcription level was not detected at this time point, on the contrary *Cbei_4000* transcription was detected only at that time.

After 5 h, when the first peak of hydrogen was observed (Fig. 3), *Cbei_1773* and *Cbei_1901* genes were expressed, respectively, 5- and 4-fold more than the genes encoding for a monomeric *Cbei_0327*, a membrane-bound *Cbei_3796* and the catalytic subunit alpha of the heterotrimeric *Cbei_4110* [FeFe]-hydrogenases (Fig. 4). At least one of the selected, monitored genes that is highly expressed in the early onset of hydrogen production (Cbei_1773) encodes for an unusual [FeFe]-hydrogenase resilient to oxygen damage [27], pointing to a specific role of this enzyme as the main hydrogen-producing catalyst, even if expressed at stages of relatively low anaerobiosis level, and therefore enhancing the tolerance of the whole system to traces of oxygen. Another intriguing hypothesis on the regulation of this gene and on the activity of its encoded protein is the inactivation observed in the protein at oxidative potentials, i.e. when the bacterial cell experiences a shortage in NAD(P)H and other reduced compounds and proteins (ferredoxins and flavodoxins) used to supply electrons. In this situation, a high concentration of an active hydrogenase might impair the redox equilibrium: although this is still a speculative hypothesis, a possible effect of the protein-based inactivation mechanism at high-potential could act as a buffering system and allow for a safer high-level expression enabling the cell to promptly reactivate the hydrogen-producing metabolism. A 99% similarity to the hydrogenase encoded by *C. beijerinckii* SM10 [27] Cbei_1773 was also observed in transcriptomic analysis [46] in the coding sequence X276_18165 from *C. beijerinckii* NRRL B-598, but the authors could not unambiguously assign to this gene the role of expressing the hydrogenase that mainly produces hydrogen in the exponential growth phase of the analysed *C. beijerinckii* NRRL B-598 strain.

After 23 h, as the hydrogen production decreased (Fig. 3), *Cbei_1773*, *Cbei_0327* and *Cbei_1901* genes were expressed, respectively, 4-fold, 1.6-fold and 1.3-fold more than the gene encoding for the catalytic subunit alpha *Cbei_4110* of the heterotrimeric [FeFe]-hydrogenases (Fig. 4). Other transcription products were not detected.

During the first 23 h of dark fermentation, transcription levels of the genes encoding for the monomeric *Cbei_1773* and the heterotrimeric *Cbei_4110* [FeFe]-hydrogenases followed the same trend observed for hydrogen production; transcription levels of the gene encoding for monomeric *Cbei_0327* showed a reverse trend compared with that of hydrogen. Expression of *Cbei_1901* was detected only after 5 and 23 h when the amount of hydrogen was different from zero in the batch; *Cbei_4000* was expressed only at the initial time point, and *Cbei_3796* expression was observed at the first two time points. The decrease of transcripts levels of all genes encoding hydrogenases can be explained partially by a stationary phase and a low transcriptional activity as highlighted by the low recA levels. A relatively high hydrogen production level has been reported in Clostridia even at low transcripts amounts, given the good stability of the [FeFe] hydrogenases as active enzymes in the cell, which could explain the relatively high hydrogen accumulation between 23 and 144 h even with low transcripts detected at 72 and 144 h.

In summary genes *Cbei_1773*, *Cbei_4110*, *Cbei_0327* and *Cbei_1901*, which undergo larger changes in expression, seem to be related to hydrogen production, although with positive (*Cbei_1773* and *Cbei_4110)* or negative (*Cbei_0327*) correlations, hence it would be interesting to further investigate their metabolic roles. In particular, the positive correlation of hydrogen production with *Cbei_1773* is relevant given the attested good productivity and the oxygen-tolerance unique feature of the hydrogenase encoded by this gene [27]. Genes *Cbei_4000* and *Cbei_3796* are possibly less related to hydrogen production and might be silent during dark fermentation. A null expression of *Cbei_4000* and negligible levels of *Cbei_3796* during hydrogen production were also observed in previous semi-quantitative experiments performed by our group in pure cultures of *C. beijerinckii* SM10 [35], although here a statistically significant change in expression (even if at a low absolute level) can be highlighted (Fig. 4). The range of different structures of the proteins encoded by the analysed genes, in terms of electron transfer domains and possible redox partners supplying or accepting electrons, covers almost completely the whole panel of classified [FeFe]-hydrogenase modular structure arrangements. The results obtained suggest that a specific physiological and functional relevance is linked to each [FeFe]-hydrogenase-encoding gene and that a differential expression in time and fine-tuning of the reciprocal amount and activity of the hydrogenases plays a key role in the entire balance of the redox equilibrium and the hydrogen metabolism in Clostridia. This could justify the redundancy and suggest an increasing evolutionary success for the strains which can rely on a broader range of different [FeFe] hydrogenases (here *C. beijerinckii* versus *C. tyrobutyricum*). The knowledge of the interplay of different hydrogenase-encoding genes is gaining importance in the study of hydrogen-producing bacteria [57], not only in Clostridia.

A summarising overview of the obtained results as for involved genes and their changes in expression level is reported in Table 1 compared to other similar studies on hydrogen-producing bacteria. Although the very different growth conditions and test strategies, the emerging landscape is that these studies are crucial to optimise the system in bioreactors and to exploit the cell and enzyme catalysts for applicative purposes. In this respect, the results of this work as for the bio-augmentation with *C. beijerinckii*, are being implemented by the project participating company AgriNewTech Srl to promote reuse of low-value compost and intermediate products (partially composted but unsuitable for completing the process) which cannot be sold on market. The outcomes also suggested economically valuable alternative exploitation of green wastes that can reduce the GHG emission, including a first dark fermentation/anaerobic digestion preliminary process and an optimised (implying low emission) composting process limited to the final digestate.

**Table 1:** Comparison of studies on [FeFe] hydrogenase genes expression in various conditions available in literature and the present study

Organism(s)	Methodological approach	Growth conditions	Focused metabolism studied	[FeFe] hydrogenase genes expression monitored	Refs.
*C. beijerinckii* SM10	Semi-quantitative RT-PCR	Clostridial medium, pure culture	Hydrogen production	**Cbei_1773** Cbei_0327 **Cbei_1901** Cbei_4110 **Cbei_3796** Cbei_4000	[35]
*C. butyricum* SM32	Semi-quantitative RT-PCR	Clostridial medium, pure culture	Hydrogen production	CBY_3049 CBY_2300 CBY_2047 **CBY_2676**	[35]
*C. perfringens* SM09	Semi-quantitative RT-PCR	Clostridial medium, pure cultures	Hydrogen production	CPF_2655 CPF_1076 **CPF_0270** CPF_2900	[35]
*C. tyrobutyricum* KCTC 5387 (ATCC 25,755)	Shotgun proteomics	2xYTG medium Pure culture	General metabolism	CTK_C05160 CTK_C26290 CTK_C29580	[43]
*Proteobacteria*, *Firmicutes*, *Bacteroidetes*, and *Chloroflexi*	DGGE analysis of *hydA in time*	Paddy Field Soil during rice straw decomposition with autochthonous microbiota	Hydrogen production	***hydA*** sequences of uncultured strains deposited to the DDBJ database under accession numbers LC041370-LC041941	[45]
*Clostridium beijerinckii* NRRL B-598	RNA-Seq	Batch fermentation, pure culture in culture broths with a glucose concentration of 50 g/L at pH 6.3	Acidogenesis, solventogenesis, metabolic stress response and life cycle changes	**X276_17350**, X276_06930 **X276_05300** (sharing high similarity with **Cbei_1901**, Cbei_3796 and **Cbei_4110**, respectively)	[46]
*Clostridium butyricum* CWBI 1009	Transcriptomic (RNA-seq and RT-qPCR) and proteomic analyses	Glucose fermentation, unregulated pH with Argon atmosphere pure culture	Hydrogen production	**hydA2,** **hydA8** **hydB2** **hydB3** (changes only observed in Argon atmosphere, not in N_2_)	[49]
*Clostridium* sp. strain H2	RT-qPCR	Under fermentation conditions, Widdel’s freshwater medium pure cultures	Hydrogen production	*H2hydA1* *H2hydA2* ***H2hydA3*** *H2hydA4* ***H2hydA5*** corresponding to *hydA* genes of *C. bifermentans* ATCC 638 (AVNC00000000) and ATCC 19,299 (AVNB00000000)	[50]
*Desulfovibrio* sp. strain A1 and AH1	RT-qPCR	Under sulphate-reducing conditions modified Widdel’s freshwater medium pure cultures	Hydrogen production	***A1hydA1*** *A1hydA2* corresponding to *hydA* genes of *D. vulgaris* Hildenborough (AE017285)	[50]
*Clostridium butyricum* CGS5	RT-PCR and qPCR	PM medium Pure culture	Hydrogen production	**h*ydA***	[51]
*Thermotoga neapolitana and Caldicellulosiruptor saccharolyticus*	**Quantitative Real-time PCR**	Synthetic co-culture	Hydrogen production	**hydA** from *Thermotoga neapolitana*	[52]
*Clostridium thermocellum* ATCC 27,405	Real-time quantitative PCR	Continuous pure culture on Modified Dehority medium containing (per litre) 3.0 g of cellobiose or 2.7 to 3.1 g of Sigmacell 20 microcrystalline cellulose	Fermentation of Cellulose or Cellobiose	**hydA**	[53]
*Clostridium beijerinckii* AM2 and *Clostridium tyrobutyricum* AM6	RT-qPCR	Green waste with autochthonous microbiota and co-addition of both strains	Hydrogen production on green waste	**Cbei_1773** **Cbei_0327** **Cbei_1901** **Cbei_4110** **Cbei_3796** Cbei_4000 Ctyr_hydA (FJ226584)	This study

The genes with changes in expression levels are highlighted in bold

## Conclusions

RT-qPCR covering the complete set of known [FeFe]-hydrogenase gene types [15, 58] was performed for the first time in a real and nutrient-poor green waste, usually employed for composting processes but re-addressable for exploitation on other markets [59] and demonstrated to be suitable as dark fermentation substrate. This latter is an advantage over composting due to complete recovery of organic compounds energy as clean fuel (bio-hydrogen or even further to CO_2_ neutral methane production) and to the demonstrated reduced emission of GHG gas (CH_4_, N_2_O) otherwise generated by the direct composting process of the green waste [34]. The matrix, containing the autochthonous microbiota was studied with the addition of a supplementary inoculum of endogenous hydrogen producers which confirmed the main role of *C. beijerinckii* in the waste-sustained hydrogen production and a good resilience of the autochthonous consortium to the bio-augmentation strategy applied. The diverse modular structure of the monitored hydrogenases is worth further investigation to evaluate if the architecture of domain arrangement is consistently related to their roles in microbial metabolism and therefore their timing of expression.

The data granted by the different aims of the paper (1: to isolate and characterise robust hydrogen producers, 2: to provide biochemical characterisation of the interplay of gene expression times/modes in a fermentation on real and complex matrices, 3: to support the enhanced use of green waste in dark fermentation rather than only for composting) are an example of how biochemical studies can support circular bioeconomy.

## Materials and methods

### Culture media

The medium used for bacteria cultures was Clostridial nutrient medium (FLUKA): agar, 0.5 g/L, L-cysteine hydrochloride, 0.5 g/L, D( +)-glucose, 5.0 g/L, meat extract, 10.0 g/L, peptone, 5.0 g/L, sodium acetate, 3.0 g/L, sodium chloride, 5.0 g/L, starch, 1.0 g/L, yeast extract, 3.0 g/L, final pH 6.8 ± 0.2 (25 °C).

Or else, minimal medium: 100 mM potassium phosphate, 17 g/l tryptone, 3 g/l peptone papaic digest of soybean, 10 g/L glucose, initial pH 7.0.

All culture media were sterilised by autoclaving for 20 min at 121 °C.

### Isolation of culturable bacteria

The cultivable microbiota present in the nutrient-poor [33] green waste (here referred as *Mix,* made by pruning of trees and a part of leaves and grass clippings collected in the province of Torino from private and public gardens) was isolated after 41 days of dark fermentation. The flask was opened and non-selective medium was added, then vital cultivable microbiota was isolated by plating of untreated dilution of *Mix* on Clostridial nutrient medium agar 1.5% w/v with dilutions from 10^–1^ to 10^–5^. From each dilution 100 µl was spread plated (two replicates) and further incubated under anaerobic conditions at 37 °C overnight. Anaerobic conditions were obtained by using Anaerogen bags (Oxoid) for the plates and fluxing argon for liquid cultures.

In order to select spore-forming microorganisms the same sample was pre-heated at 85 °C for 20 min prior to dilution from 10^–1^ to 10^–5^ before plating. In the untreated sample, 2.1 × 10^5^ CFU/mL and in the pre-heated sample 1.6 × 10^4^ CFU/mL were counted. Following incubation, single and isolated colonies with different morphologies were randomly selected and pure cultures were obtained by an additional passage on plate. Every 4 days, each strain was replicated in a new plate in anaerobic conditions to maintain it viable.

A total of 31 single colonies, 18 from the untreated and 13 from the pre-heated samples, were selected based on the colony morphology for further identification and characterisation. All the isolates were classified based on cell morphology after Gram staining and growth ability also in the presence of oxygen, thus discriminating oxygen-tolerant from oxygen-sensitive microorganisms.

### Morphological characterisation

Gram staining (Fluka kit) was performed to analyse the morphology of the bacteria, which were transferred from fresh plates and spread over a drop of water onto the surface of a clean glass slide. A tenfold dilution was made to the drop of microorganism, which was then placed on a glass slide using an inoculation loop and dried with a Bunsen flame for a few seconds. The slide was then flooded with the Gram’s crystal violet solution (Fluka) for 1 min at most and then removed with water. Gram’s iodine solution (Fluka) was used as mordant, applied for 1 min and washed away again with water. After that, the glass slide was covered with Gram’s decolouriser solution (Fluka) for 20 s followed by washing and flooding with Gram’s safranin solution (Fluka) for 1 min and then washed once again. Morphological examinations were performed with a Reichert microscope. Glass slides were observed through optic microscope at 1000 × magnification using immersion oil.

### Genomic DNA extraction and 16S rRNA-encoding gene amplification

For identification, each isolated strain consisted of bacteria picked up from fresh plates (incubated under anaerobic conditions at 37 °C overnight) using a sterile loop and it was suspended into 70 μL of sterile Milli-Q water. DNA extraction was performed by 3 cycles of freeze/thawing at 90 °C/liquid nitrogen and centrifuged for 10 min at max speed. Supernatant was recovered and diluted with 200 μL of sterile water.

The 16S rRNA-encoding gene was amplified from genomic DNA by PCR using the proof-reading polymerase “KOD Hot Start DNA polymerase” (Merck Millipore) following the manufacturer's instructions and the two universal primers [44, 51]: 27F: 5’-AGAGTTTGATYMTGGCTCAG-3’, 1492R: 5’-TACGGYTACCTTGTTACGACT-3'.

PCR fragments were separated on 1.5% agarose gel (TAE 1X) using PerfectSize DNA molecular weight 1 kb XL ladder as reference (5Prime). The PCR product was purified using Nucleo spin gel and PCR clean-up kit (Macherey-Nagel) following the manufacturer's instructions.

### Restriction fragment length polymorphism (RFLP) analysis of 16S rRNA-encoding genes

The amplified DNA was then digested using four endonuclease restriction enzymes *Alu*I, *Hae*III, *Hha*I and *Taq*I (Fermentas) separately, in order to obtain RFLP fingerprinting.

The amplified 16S rRNA-encoding gene products were digested following the manufacturer’s instructions. DNA fragments were separated by electrophoresis on 1.5% w/v agarose gel 1X TAE stained with SYBR® Safe (Invitrogen) for 1 h at 100 V, 400 mA. The reference used was PerfectSize DNA molecular weight 100 bp XL Ladder (5 Prime). Scanned images of the gels containing DNA-RFLP were captured with Quantity One software (Bio-Rad).

### Sequencing of 16S rRNA-encoding genes and phylogenetic analysis

After classification, one isolate per group was selected. The 16S rRNA-encoding gene was amplified by PCR as described above, purified with a PCR clean-up kit (Macherey-Nagel) and the concentration was evaluated with NanoVue instrument (GE Healthcare).

The DNA amplified and purified was diluted to a final concentration of 10 μg/mL in a volume of 15 μL and then sequenced. The sequencing was carried out by Eurofins MWG Operon Company. The sequences obtained were searched against NCBI, EMBL and DDJB databases using BLASTN 2.0.5. The GenBank accession number of the 16S rRNA-encoding gene sequences are reported below:

group A: MZ054377, group B: MZ054378, group C: MZ054379, group D: MZ054380, group E: MZ054381, group F: MZ054382, group G: MZ054383, group H: MZ054384, group I: MZ054385, group K: MZ054386.

The identity ≥ 99% with e-value of 0.0 was found for each species.

Phylogenetic trees were constructed by neighbour-joining methods using Molecular Evolutionary Genetics Analysis (MEGA) version 6.06 [60]. The topology of the tree was evaluated by means of bootstrap analysis based on 1000 replicates.

### Quantification of H_2_ and CH_4_ production

Pure cultures were grown in a 20-mL glass vial sealed with a butyl rubber stopper, containing 4 mL of sterile minimal medium under anaerobic argon atmosphere at 37 °C, 250 rpm for 16 or 24 h. The negative control was sterile medium without bacteria inoculation. Experiments with the green waste biomass were performed as described previously [33]. The gas was sampled with a syringe SampleLock Gastight syringe (Hamilton) and analysed by a gas chromatographer (Agilent 7890A) with TCD detector. The device was equipped with purged packed inlet, HP-Molesieve 5A column (length 30 m, 0.530 mm ID, film 25 mM). Argon, used as carrying gas, had a flow of 0.879 mL/min. Efficient and quantitative separation was achieved in 2.8 min at 60 °C, the gas chromatography method allowed the detection of H_2_, O_2_, N_2_, and CH_4_, respectively, at 1.4, 1.6, 1.9 and 2.4 min. The gas chromatographer injector temperature was 60 °C and the detector temperature was 250 °C. H_2_ and CH_4_ quantifications were obtained by calibration curves prepared from standard gases (Rivoira, Italy).

### Green waste bio-augmented with hydrogen-producing *C. beijerinckii* AM2 and *C. tyrobutyricum* AM6 isolates

For the pre-cultures preparation, anaerobic sterile medium was inoculated with *C. beijerinckii* AM2 or *C. tyrobutyricum* AM6 and incubated at 37 °C, 180 rpm for 16 h. For culture preparation 1 mL of the pre-culture was inoculated in 40 mL of anaerobic and sterile medium and incubated at 37 °C 180 rpm for 16 h. In order to minimise contamination and thoroughly analyse the sample in its original characteristics, the same spring green waste was divided into sterile glass vials with a volume of 60 ml. Each vial containing 5 g of green waste was sealed with butyl rubber stoppers and flushed with argon for 25 min. The bacteria *C. beijerinckii* 5 × 10^5^ CFU (1 mL) and *C. tyrobutyricum* 5 × 10^6^ CFU were inoculated in each vial by means of a sterile syringe in a sequential manner. All the samples were incubated at 37 °C, 220 rpm. Ten time points were selected and the gas samples were taken, respectively, after 0, 5, 23, 23, 42, 48, 72, 144, 216 and 288 h from the initial time of incubation. The control group used was the very same spring green waste just without *C. beijerinckii* and *C. tyrobutyricum* inoculation and the same volume of sterile medium was added to the non-augmented mixture to check real-time performances and compare hydrogen production over the long run. The data collected correspond to the average of two independent samples (biological replicates) analysed at least in triplicate.

### RNA extraction and purification

Metal beads and all glassware used in the isolation of total RNA from green waste were kept at 180 °C overnight; plasticware was autoclaved before use, whereas solutions used in RNA extractions were treated with 0.1% (v/v) diethylpyrocarbonate (DEPC) and autoclaved to inactivate RNases. Two samples per time point were collected immediately. Each sample (100 mg) was homogenised in liquid nitrogen with metal beads using TissueLyserII, then the RLT extraction buffer was added and the tube was incubated at 56 °C for 1–3 min. Total RNA was extracted and purified in triplicate from the pooled samples following using the kit “Rneasy Plant Mini Kit” (Qiagen). The total RNA was treated with LiCl (3 M). The sample was incubated at 4 °C on ice overnight and then RNA was selectively pelleted after centrifugation at 21,000 g for 30 min at 4 °C. The pellet was washed with cold ethanol (70%), dried and re-suspended in DEPC–water. The total RNA was treated with Ambion® TURBO DNA-free DNase (Ambion, Life Technologies), according to the manufacturer’s instructions. RNA purity and concentration were assessed using a NanoDrop 1000 spectrophotometer (Thermo Scientific, Wilmington, DE, USA) by determining the spectrophotometric absorbance of the samples at 230, 260 and 280 nm and ratios of A_260_:A_280_ and A_260_:A_230_ (A260/280 ≥ 2 and A260/230 ≥ 1.8). The RNA integrity was evaluated from the 23S and 16S rRNA bands on 1.2% agarose gel after electrophoresis, staining with SYBR® Safe (Invitrogen). The absence of gDNA contamination was tested by omitting reverse transcriptase and testing PCR reaction using specific primers for *recA* genes by ONE STEP kit (Sigma).

### Primers design, reference gene and standard curves preparation

The specific *recA* gene is used as an internal reference gene in reverse RT-qPCR. No *recA* gene of *C. tyrobutyricum* was reported in the NCBI database so, from gDNA of *C. beijerinckii* AM2 and *C. tyrobutyricum* AM6 *recA* genes were amplified with degenerate primers for *Clostridium sp*: RecA-F1: 5’-GATGCNGARCATGCNYTNGA-3’, RecA-R1: 5’-CATTYTCHCKWCCYTGDCCWA-3’ (N = A, C, T, G; R = A, G; Y = C, T; H = A, C, T; K = C, T; D = A, T, G; W = A, T), which generated a fragment of around 634 bp [58].

The obtained sequences (GenBank accession numbers: *C. beijerinckii* AM2 recA: MZ062441, *C. tyrobutyricum* AM6 recA: MZ062442) were used to design new RT-qPCR primer pairs for *recA* that were specific for *C. beijerinckii* and *C. tyrobutyricum.* The new *recA* primer pairs were designed by Primer3 tool to obtain amplification products that were no longer than 200 bp, as well as cover regions that were conserved within a species and were as different as possible from the other affiliated species. Specificity and cross-reactivity were tested using BLAST (NCBI).

For each hydrogenase gene between 5 and 10 *hydA* gene sequences encoding for the same structure type hydrogenase from the NCBI (National Center for Biotechnology Information) database were aligned using ClustalW to form 5’–3’ consensus sequences. These were used to develop the specific primer sets by AnnHyb tool. The selected sets were synthesised by Eurofins MWG (Germany). The amplicon fragment sizes ranged from 161 to 111 bp as reported in Table 2.

**Table 2:** List of primers, amplicon fragment size and features

Gene description	Forward (5′-3′)	Reverse (5′-3′)	Size (bp)	E%	R2	Tm (℃)
*Ctyr_recA*	GTGGAAGCATTGGTTAGATC	GTCCTACATGAGAATCTCCCA	111	101	0.98	75.1
*Cbei_recA*	GTCACAGGCGTTAAGAAAGCT	TGCTCTTCCTCCAGTTGTTG	129	87.5	0.99	74.5
*Cbei_1773*	GAGGTTGGGATGGATTCAGA	CCACCTACACACGCCATTA	161	89.3	0.99	75.6
*Cbei_0327*	AGCCTTATTCGATGCGTTTG	TCAATCCACCACCTACAGCA	104	95.9	0.99	74.8
*Cbei_1901*	TTGTGGTGTATGCGTGGATT	AGTACCAAATTGCCCGCTA	139	88.0	0.99	74.4
*Cbei_4000*	AGGGTGGCATAAATGGAGGTG	CCTCTGCCCTTCCTCTCTAACA	140	97.1	0.99	75.3
*Cbei_4110*	TGGTGATTGATGGCAATAGG	TCATCCTCGACCACACACAT	148	95.2	0.99	73.3
*Cbei_3796*	TGTTTGCGTTTCTTGTGGAC	TGGCTCCTTCACACTCTCAA	127	103	0.98	72.7
*Ctyr_hydA′*	CCATGCCCAAGAAGAGAAAA	GCATTTGGTTCTGTCAAGCA	144	88.0	0.99	75.0
*Ctyr_hydA″*	GAGGCAAATGGCAGAACAAT	TTTTCTCTTCTTGGGCATGG	161	95.2	0.99	74.7

The complete sequencing of the selected hydrogenases genes was performed. Reference numbers of hydrogenases sequences containing silence or missense mutations and deposited in GenBank are: *C. beijerinckii* AM2 1773: MZ062438, *C. beijerinckii* AM2 1901: MZ062439 and *C. tyrobutyricum* AM6 hydA: MZ062440.

The amplicon sequences were screened using BLAST to determine cross-reactivity.

The specificity for all primer pairs were experimentally evaluated with a range of target and non-target *Clostridium* species. The primers targeting the *recA* and *hyd* genes were specific (100% identity) also when used in RT-qPCR optimisation. No amplification for negative controls was observed. The high specificity of the designed oligonucleotides and their amplification conditions was demonstrated at the specific melting temperatures reported in Table 2, conditions under which all the genes tested showed positive signals. The expected fragment (Table 2) gave a single band when visualised on a 2% agarose gel. To determine the detection limits, reaction efficiencies and linear ranges of amplification, the standard curves were generated using genomic DNA as reported in Yun et al. [61], DNA was extracted and purified from pure cultures by Wizard genomic DNA purification kit (Promega). Seven tenfold dilutions were freshly prepared each time from the DNA, ranging from 15 to 15 X 10^–5^ ng/µl. The good linearity is demonstrated by linear correlation coefficient (R^2^) value ≥ 0.98 for six orders of magnitude for all the genes and the slope of the regression curve showed efficient yields ranging between 101 and 87.5% the values for each primers couple are reported in Table 2.

### cDNA synthesis and real-time qPCR

The first-strand cDNA was synthesised starting from 0.645 µg of total RNA by using a selected iScript Reverse Transcriptase (Invitrogen) following the manufacturer’s instructions. The analysis was performed in 96-well plate using SYBR Green method (Power SYBR® Green PCR Master Mix, Applied Biosystems). The PCR reaction mix contained 1 µL of cDNA, 0.2 mM dNTPs, 0.2 µM each primer, 1.5 mM MgCl_2_ and 0.5 unit Taq polymerase. Cycling conditions consisted of an initial denaturation phase at 95 °C for 10 min, followed by 40 cycles at 95 °C for 15 s and 60 °C for 1 min. At the end of each RT-qPCR run, a melting analysis was carried out to verify the absence of non-specific amplification with 95 °C for 15 s, 60 °C for 1 min and 95 °C for 15 s with a transition rate of 0.3 °C every 10 s. Non-RT controls (using total RNA without reverse transcription to monitor for genomic DNA contamination) and non-template controls (water instead of template) were included in all runs. Gene expression was determined as the mean, and standard errors were calculated over all biological and technical replicates. The standard curve was generated by performing three independent serial dilutions of the DNA standard and by assaying each dilution in duplicate together with negative control reactions. In order to calculate the copy number of each target gene in the samples the standard curve method was used and the hydrogenase gene expression was normalised on the total RNA and later on the reference gene expression.

## Data Availability

All data generated or analysed during this study are included in this published article.

## References

[1] Abreu A, Alves JI, Pereira MA, Karakashev D, Alves MM, Angelidaki I (2010). Engineered heat treated methanogenic granules: a promising biotechnological approach for extreme thermophilic biohydrogen production. Bioresour Technol.

[2] Zhao X, Xing D, Fu N, Liu B, Ren N (2011). Hydrogen production by the newly isolated *Clostridium beijerinckii* RZF-1108. Bioresour Technol.

[3] Jung KW, Kim DH, Kim SH, Shin HS (2011). Bioreactor design for continuous dark fermentative hydrogen production. Bioresour Technol.

[4] Kim TH, Lee Y, Chang KH, Hwang SJ (2012). Effects of initial lactic acid concentration, HRTs, and OLRs on bio-hydrogen production from lactate-type fermentation. Bioresour Technol.

[5] Barca C, Soric A, Ranava D, Giudici-Orticoni MT, Ferrasse JH (2015). Anaerobic biofilm reactors for dark fermentative hydrogen production from wastewater: a review. Bioresour Technol.

[6] Kumar G, Sivagurunathan P, Park JH, Park JH, Park HD, Yoon JJ, Kim SH (2016). HRT dependent performance and bacterial community population of granular hydrogen-producing mixed cultures fed with galactose. Bioresour Technol.

[7] Kumar G, Shobana S, Nagarajan D, Lee DJ, Lee KS, Lin CY, Chen CY, Chang JS (2018). Biomass based hydrogen production by dark fermentation-recent trends and opportunities for greener processes. Curr Opin Biotechnol.

[8] Wang J, Bibra M, Venkateswaran K, Salem DR, Rathinam NK, Gadhamshetty V, Sani RK (2018). Biohydrogen production from space crew's waste simulants using thermophilic consolidated bioprocessing. Bioresour Technol.

[9] Rabelo CABS, Soares LA, Sakamoto IK, Silva EL, Varesche MBA (2018). Optimization of hydrogen and organic acids productions with autochthonous and allochthonous bacteria from sugarcane bagasse in batch reactors. J Environ Manage.

[10] Yang G, Wang J (2019). Changes in microbial community structure during dark fermentative hydrogen production. Int J Hydrog Energy.

[11] Xiao Y, Zhang X, Zhu M, Tan W (2013). Effect of the culture media optimization, pH and temperature on the biohydrogen production and the hydrogenase activities by *Klebsiella pneumoniae* ECU-15. Bioresour Technol.

[12] Zahedi S, Solera R, Micolucci F, Cavinato C, Bolzonella D (2016). Changes in microbial community during hydrogen and methane production in two-stage thermophilic anaerobic co-digestion process from biowaste. Waste Manag.

[13] Tolvanen KES, Korskinen PEP, Ylikoski A, Hemmila O, Puhakka J, Karp MT (2008). Quantitative monitoring of a hydrogen-producing *Clostridium butyricum* strain from a continuous-flow, mixed culture bioreactor employing real-time PCR. Int J Hydrog Energy.

[14] Vignais PM, Billoud B (2007). Occurrence, classification, and biological function of hydrogenases: an overview. Chem Rev.

[15] Calusinska M, Happe T, Joris B, Wilmotte A (2010). The surprising diversity of clostridial hydrogenases: a comparative genomic perspective. Microbiology.

[16] Therien JB, Artz JH, Poudel S, Hamilton TL, Liu Z, Noone SM, Adams MWW, King PW, Bryant DA, Boyd ES, Peters JW (2017). The physiological functions and structural determinants of catalytic bias in the [FeFe]-hydrogenases CpI and CpII of *Clostridium pasteurianum* strain W5. Front Microbiol.

[17] Nicolet Y, Piras C, Legrand P, Hatchikian CE, Fontecilla-Camps JC (1999). *Desulfovibrio desulfuricans* iron hydrogenase: the structure shows unusual coordination to an active site Fe binuclear center. Structure.

[18] Peters JW, Lanzilotta WN, Lemon BJ, Seefeldt LC (1998). X-ray crystal structure of the Fe-only hydrogenase (CpI) from *Clostridium pasteurianum* to 1.8 Angstrom resolution. Science.

[19] Demuez M, Cournac L, Guerrini O, Soucaille P, Girbal L (2007). Complete activity profile of *Clostridium acetobutylicum* [FeFe]-hydrogenase and kinetic parameters for endogenous redox partners. FEMS Microbiol Lett.

[20] Happe T, Naber JD (1993). Isolation, characterization and N-terminal amino acid sequence of hydrogenase from the green alga *Chlamydomonas reinhardtii*. Eur J Biochem.

[21] Caserta G, Adamska-Venkatesh A, Pecqueur L, Atta M, Artero V, Roy S, Reijerse E, Lubitz W, Fontecave M (2016). Chemical assembly of multiple metal cofactors: the heterologously expressed multidomain [FeFe]-hydrogenase from *Megasphaera elsdenii*. Biochim Biophys Acta.

[22] Chongdar N, Birrell JA, Pawlak K, Sommer C, Reijerse EJ, Rüdiger O, Lubitz W, Ogata H (2018). Unique spectroscopic properties of the H-cluster in a putative sensory [FeFe] hydrogenase. J Am Chem Soc.

[23] Engelbrecht V, Rodríguez-Maciá P, Esselborn J, Sawyer A, Hemschemeier A, Rüdiger O, Lubitz W, Winkler M, Happe T (2017). The structurally unique photosynthetic *Chlorella variabilis* NC64A hydrogenase does not interact with plant-type ferredoxins. Biochim Biophys Acta.

[24] Greene BL, Schut GJ, Madams MWW, Dyer BR (2017). Pre-steady-state kinetics of catalytic intermediates of an [FeFe]-hydrogenase. ACS Catal.

[25] Land H, Ceccaldi P, Mészáros LS, Lorenzi M, Redman HJ, Senger M, Stripp ST, Berggren G (2019). Discovery of novel [FeFe]-hydrogenases for biocatalytic H 2-production. Chem Sci.

[26] Morra S, Valetti F, Sarasso V, Castrignanò S, Sadeghi SJ, Gilardi G (2015). Hydrogen production at high Faradaic efficiency by a bio-electrode based on TiO_2_ adsorption of a new [FeFe]-hydrogenase from *Clostridium perfringens*. Bioelectrochemistry.

[27] Morra S, Arizzi M, Valetti F, Gilardi G (2016). Oxygen stability in the new [FeFe]-hydrogenase from *Clostridium beijerinckii* SM10 (CbA5H). Biochemistry.

[28] Morra S, Mongili B, Maurelli S, Gilardi G, Valetti F (2016). Isolation and characterization of a new [FeFe]-hydrogenase from *Clostridium perfringens*. Biotechnol Appl Biochem.

[29] Pan CM, Fan YT, Zhao P, Hou HW (2008). Fermentative hydrogen production by the newly isolated *Clostridium beijerinckii* Fanp3. Int J Hydrogen Energy.

[30] Polliotto V, Morra S, Livraghi S, Valetti F, Gilardi G, Giamello E (2016). Electron transfer and H_2_ evolution in hybrid systems based on [FeFe]-hydrogenase anchored on modified TiO_2_. Int J Hydrog Energy.

[31] Tomazetto G, Wibberg D, Schlüter A, Oliveira VM (2015). New FeFe-hydrogenase genes identified in a metagenomic fosmid library from a municipal wastewater treatment plant as revealed by high-throughput sequencing. Res Microbiol.

[32] Land H, Sekretareva A, Huang P, Redman HJ, Németh B, Polidori N, Mészáros LS, Senger M, Stripp ST, Berggren G (2020). Characterization of a putative sensory [FeFe]-hydrogenase provides new insight into the role of the active site architecture. Chem Sci.

[33] Arizzi M, Morra S, Pugliese M, Gullino ML, Gilardi G, Valetti F (2016). Biohydrogen and biomethane production sustained by untreated matrices and alternative application of compost waste. Waste Manag.

[34] Zhu-Barker X, Bailey SK, Paw UKT, Burger M, Horwath WR (2017). Greenhouse gas emissions from green waste composting windrow. Waste Manag.

[35] Morra S, Arizzi M, Allegra P, La Licata B, Sangnelli F, Zitella P, Gilardi G, Valetti F (2014). Expression of different types of [FeFe]-hydrogenase genes in bacteria isolated from a population of a biohydrogen pilot-scale plant. Int J Hydrog Energy.

[36] Tohno M, Kobayashi H, Tajima K, Uegaki R (2012). Strain-dependent effects of inoculation of *Lactobacillus plantarum* subsp. plantarum on fermentation quality of paddy rice (*Oryza sativa* L. subsp. japonica) silage. FEMS Microbiol Lett.

[37] Ten LN, Wan-Taek IM, Sang-Hoon B, Jung-Sook L, Hee-Mock O, Sung-Taik L (2006). *Bacillus ginsengihumi* sp. nov., a novel species isolated from soil of a ginseng field in Pocheon province, South Korea. J Microbiol Biotechnol.

[38] Valdez-Vazquez I, Morales AL, Escalante AE (2017). History of adaptation determines short-term shifts in performance and community structure of hydrogen-producing microbial communities degrading wheat straw. Microb Biotechnol.

[39] Jia X, Xi B-D, Li M-X, Yang Y, Wang Y (2017). Metaproteomics analysis of the functional insights into microbial communities of combined hydrogen and methane production by anaerobic fermentation from reed straw. PLoS ONE.

[40] Gomez-Romero J, Gonzalez-Garcia A, Chairez I, Torres L, García-Peña EI (2014). Selective adaptation of an anaerobic microbial community: biohydrogen production by co-digestion of cheese whey and vegetables fruit waste. Int J Hydrog Energy.

[41] Noparat P, Prasertsan P, O-Thong S (2011). Isolation and characterization of high hydrogen-producing strain *Clostridium beijerinckii* PS-3 from fermented oil palm sap. Int J Hydrog Energy.

[42] Jo JH, Jeon GO, Lee SY, Lee DS, Park JM (2010). Molecular characterization and homologous overexpression of [FeFe]-hydrogenase in *Clostridium tyrobutyricum* JM1. Int J Hydrogen Energy.

[43] Lee J, Jang YS, Han MJ, Kim JY, Lee SY. Deciphering *Clostridium tyrobutyricum* metabolism based on the whole-genome sequence and proteome analyses. MBio 2016;7: e00743–16. 10.1128/mBio.00743-16 PMC4916380 27302759

[44] Wang X, Hoefel D, Saint CP, Monis PT, Jin B (2007). The isolation and microbial community analysis of hydrogen producing bacteria from activated sludge. J Appl Microbiol.

[45] Baba R, Asakawa S, Watanabe T (2016). H_2_-producing bacterial community during rice straw decomposition in paddy field soil: estimation by an analysis of [FeFe]-hydrogenase gene transcripts. Microbes Environ.

[46] Patakova P, Branska B, Sedlar K, Vasylkivska M, Jureckova K, Kolek J, Koscova P, Provaznik I (2019). Acidogenesis, solventogenesis, metabolic stress response and life cycle changes in *Clostridium beijerinckii* NRRL B-598 at the transcriptomic level. Sci Rep.

[47] Sedlar K, Koscova P, Vasylkivska M, Branska B, Kolek J, Kupkova K, Patakova P, Provaznik I (2018). Transcription profiling of butanol producer *Clostridium beijerinckii* NRRL B-598 using RNA-Seq. BMC Genomics.

[48] Vasylkivska M, Jureckova K, Branska B, Sedlar K, Kolek J, Provaznik I, Patakova P (2019). Transcriptional analysis of amino acid, metal ion, vitamin and carbohydrate uptake in butanol-producing *Clostridium beijerinckii* NRRL B-598. PLoS ONE.

[49] Calusinska M, Hamilton C, Monsieurs P, Mathy G, Leys N, Franck F, Joris B, Thonart P, Hiligsmann S, Wilmotte A (2015). Genome-wide transcriptional analysis suggests hydrogenase- and nitrogenase-mediated hydrogen production in *Clostridium butyricum* CWBI 1009. Biotechnol Biofuels.

[50] Baba R, Morita M, Asakawa S, Watanabe T (2017). Transcription of [FeFe]-hydrogenase genes during H2 production in *Clostridium* and *Desulfovibrio* spp. isolated from a paddy field soil. Microbes Environ.

[51] Wang MY, Tsai YL, Olson BH, Chang JS (2008). Monitoring dark hydrogen fermentation performance of indigenous *Clostridium butyricum* by hydrogenase gene expression using RT-PCR and qPCR. Int J Hydrog Energy.

[52] Okonkwo O, Lakaniemi A-M, Santala V, Karp M, Mangayil R (2018). Quantitative real-time PCR monitoring dynamics of *Thermotoga neapolitana* in synthetic co-culture for biohydrogen production. Int J Hydrog Energy.

[53] Stevenson DM, Weimer PJ (2005). Expression of 17 genes in Clostridium thermocellum ATCC 27405 during fermentation of cellulose or cellobiose in continuous culture. Appl Environ Microbiol.

[54] Savichtcheva O, Joris B, Wilmotte A, Calusinska M (2011). Novel FISH and quantitative PCR protocols to monitor artificial consortia composed of different hydrogen-producing Clostridium spp. Int J Hydrog Energy.

[55] Gomes AÉI, Stuchi LP, Siqueira NMG, Henrique JB, Vicentini R, Ribeiro ML, Darrieux M, Ferraz LFC (2018). Selection and validation of reference genes for gene expression studies in Klebsiella pneumoniae using Reverse Transcription Quantitative real-time PCR. Sci Rep.

[56] Chang JJ, Chou C, Hsu P, You S, Chen W, Lay J (2007). Flow-FISH analysis and isolation of clostridial strains in anaerobic semi-solid biohydrogen producing system by hydrogenase gene target. Appl Microbiol Biotechnol.

[57] Greening C, Biswas A, Carere CR, Jackson CJ, Taylor MC, Stott MB, Cook GM, Morales SE (2016). Genomic and metagenomic surveys of hydrogenase distribution indicate H2 is a widely utilised energy source for microbial growth and survival. ISME J.

[58] Calusinska M, Joris B, Wilmotte A (2011). Genetic diversity and amplification of different clostridial [FeFe] hydrogenases by group-specific degenerate primers. Lett Appl Microbiol.

[59] De Corato U, De Bari I, Viola E, Pugliese M (2018). Assessing the main opportunities of integrated biorefining from agro-bioenergy co/by-products and agroindustrial residues into high-value added products associated to some emerging markets: a review. Renew Sustain Energy Rev.

[60] Tamura K, Stecher G, Peterson D, Filipski A, Kumar S (2013). MEGA6: molecular evolutionary genetics analysis version 6.0. Mol Biol Evol.

[61] Yun JJ, Heisler LE, Hwang IIL, Wilkins O, Lau SK, Hyrcza M, Jayabalasingham B, Jin J, McLaurin J, Tsao M, Der SD (2006). Genomic DNA functions as a universal external standard in quantitative real-time PCR. Nucleic Acids Res.

